# Prevalence of Hypothyroidism Among Carpal Tunnel Syndrome Patients at a Hospital in Saudi Arabia

**DOI:** 10.7759/cureus.12264

**Published:** 2020-12-25

**Authors:** Faris Aldaghri, Mohammed S Algahtani, Talal A Almutairi, Moath Albusair, Khalid Bin Ghali, Fahad S Al Asim

**Affiliations:** 1 Plastic Surgery, Imam Muhammad Ibn Saud Islamic University, Riyadh, SAU

**Keywords:** prevalence, hypothyroidism, carpal, tunnel, syndrome

## Abstract

Introduction

Patients with carpal tunnel syndrome have a high prevalence of hypothyroidism, therefore, it is recommended to assess thyroid function routinely in patients with carpal tunnel syndrome. This study aims to determine the prevalence of hypothyroidism among carpal tunnel patients and to relate carpal tunnel in hypothyroidism to other socio-demographic factors.

Methods

This was a retrospective study carried out in King Fahad Medical City (KFMC) in Riyadh, Saudi Arabia. A total of 422 samples were collected conveniently from the files of patients who underwent carpal tunnel release surgery. The Statistical Package for the Social Sciences (SPSS) version 23 was used for data processing. The chi-square test was used to test the association between the categorical variables. A p-value of less than .05 was considered to be significant.

Results

Most of the respondents were females and most of them within the age group of 46 to 60 years. Thirteen point eight percent (13.8%) of the respondents are suffering from hypothyroidism and 5% from the sub-clinical form of the disease. More than two-thirds of those with hypothyroidism were asymptomatic. The duration of the disease was one to five years, and bilateral nerve concerns were mostly present in patients with carpal tunnel syndrome. The presence of thyroid abnormality doesn’t affect the duration of carpal tunnel syndrome but body mass index (BMI) is significantly associated with hypothyroidism (p-value = .001).

Conclusion

Clinical symptoms of hypothyroidism are mostly absent in patients with carpal tunnel syndrome. Most patients with carpal tunnel syndrome have the disease for one to five years and this is not significantly associated with abnormal thyroid. Most patients have bilateral wrist involvement with no apparent symptoms and signs.

## Introduction

A principal reason for carpal tunnel syndrome (CTS) is occupational and personal factors. CTS is characterized by a noticeable work disability [1]. The only treatment CTS necessitates is a surgical treatment because there are limited results with non-surgical treatments [2]. Patients with CTS show a great prevalence of hypothyroidism. Hypothyroidism showed its first association with CTS in 1954 when a patient with CTS was checked for myxedema [3]. There is a variation between the relationship of CTS with hypothyroidism ranging from 7% to 92% [4]. Moreover, according to the British Society for Surgery of the Hand (BSSH), the requirement of screening CTS patients is considered to be necessary for thyroid dysfunction [5]. Hypothyroid patients can improve their quality of life by thyroid replacement therapy. However, it is uncertain that thyroid replacement therapy can treat CTS symptoms too. Some studies have reported that thyroid replacement therapy treats symptoms of CTS only in the case of hypothyroid patients. But there are no studies about the association of biochemically corrected hypothyroidism with CTS [6].

CTS is caused by damage to the median nerve and pressure on the carpal tunnel. There are various reasons for this medical condition, which include blood vessel stenosis, pressure in the wrist area, and unnecessary use of the wrist [6]. The advanced level of hypothyroidism is known as myxedema in which little or no thyroid hormone is produced. Women >60 years of age show more prevalence of hypothyroidism as compared to men [7]. In various studies, thyroid patients have also shown a prevalence of CTS. Multiple studies have reported that numbness, tingling, and paresthesia in CTS patients occurs due to anemia or hyperlipidemia [8]. Sometimes, in subcutaneous tissues, mucopolysaccharides, hyaluronic acid, and glycosaminoglycans excessively deposit. This leads to dermal edema in hypothyroidism. There is a narrow gap in the carpal tunnel in which pseudo-mucinous substances deposit, which results in the compression of the median nerve and leads to CTS [9].

In the U.S. population, there is a 7%-12% prevalence of wrist pain and CTS. The adult population shows a 1.6% prevalence of the symptoms of CTS such as upper extremity functional impairment, weakness of hand and wrist, and paresthesia [10]. Aside from hypothyroidism, CTS is also associated with acromegaly, hemodialysis, hyperthyroidism, and carpal stenosis. In Saudi Arabia, the prevalence of CTS is 6%-15% [11]. However, a few studies have been reported to show the association between hypothyroidism and CTS. In current evidence, Saudi women show a prevalence of hypothyroidism more than Saudi men. Four out of 1000 women population are diagnosed with hypothyroidism [12]. In hypothyroidism, patients with CTS also experience abnormality in the peripheral nerves but the information in this regard is vague. Abnormality in the peripheral nerves occurs due to the excess deposition of mucinous substances, which causes axonal degeneration. If left untreated, it leads to mononeuropathy and polyneuropathy [13]. Various studies report that the nerve conduction of hypothyroid patients leads to 29% chances of being diagnosed with CTS [7,13]. The only choice for treating carpal tunnel syndrome is the decompression of the median nerve, which highly depends on postoperative care and the training of surgeons [14].

Radioactive iodine-induced hypothyroidism is also an unusual risk factor for bilateral carpal tunnel syndrome (BCTS), which is medically treatable. Several studies have reported that if a patient with hypothyroidism develops BCTS and hypertension, both these conditions can be treated [9]. Many research studies also provide evidence of the association of hypothyroidism with neuromuscular dysfunction. Peripheral neuropathy and sensorimotor polyneuropathy, common in CTS, patients have also shown an association with hypothyroidism [15-16]. The prevalence of polyneuropathy in both CTS and hypothyroidism is 40%-73%. Hypothyroidism as a cause of CTS and polyneuropathy shows a prevalence of 4%-5% [16].

There is a need for the treatment of CTS in patients with treated or untreated hypothyroidism to prevent both disorders; otherwise, patients can also experience cardiovascular and respiratory system [15]. Early detection and treatment are necessary because untreated CTS can cause many nerve complications. The diagnosis of CTS patients with a prevalence of hypothyroidism can be ensured by communicating with primary and secondary healthcare providers [5]. Only a few studies have clearly determined hypothyroidism as a risk factor for CTS patients. Moreover, the paraclinical symptoms of CTS in female populations of different countries are more as compared to male populations. In Saudi Arabia, no such study is available on the importance of the early detection of hypothyroidism in CTS patients. More sensitive and better standards should be developed for detecting symptoms of carpal tunnel syndrome in patients with hypothyroidism. This study aims to identify the prevalence of hypothyroidism among CTS patients in Saudi Arabia with more efficient and valued treatments.

As advised by the British Society for the Surgery of Hand (BSSH), patients with CTS must be screened for hypothyroidism before treatment. Many studies have reported the association of hypothyroidism with CTS through screening processes. One such study was conducted by Vashishtha et al. (2016) who aimed to assess CTS patients to observe the association of CTS with hypothyroidism. In a UK teaching hospital, patients from the past three years who were treated surgically were assessed. Their medical records and pathology were also assessed. One-hundred patients with CTS were involved in this retrospective study. The results showed that three out of 100 patients were diagnosed with preoperative thyroid diseases. Two out of three patients had hypothyroidism. The results showed that CTS patients showed a prevalence of preoperative hypothyroidism [5].

Another study was conducted by Shiri (2014) in which the magnitude of hypothyroidism and CTS was assessed. This meta-analysis included many different studies. These studies confirmed CTS cases with nerve conduction. Ten studies estimated that there is a link between CTS and hypo or hyperthyroidism. There was a 1.32 pooled effect size in those 10 studies. The results showed that there was strong strength between surgery for CTS and hypothyroidism as compared to the CTS and hypothyroidism association. Moreover, it was also reported in this study that CTS was a risk factor in hypothyroidism patients because pseudo-mucinous materials were deposited in the median nerve of patients. Tendons in the carpal tunnel can also be thickened and swollen in case of hypothyroidism. The patients undergoing thyroid replacement therapy can also experience CTS symptoms [17].

As hypothyroidism is more prevalent in CTS female patients, a study was conducted to determine the prevalence of CTS in female patients with hypothyroidism. This study included 300 female hypothyroid patients who were provided with the Boston Carpal tunnel syndrome questionnaire. For each patient, the compression, Phalen, and Tinel’s tests were assessed. Electrodiagnostic testing was also performed on the basis of which the patients were categorized into four groups: normal, patients with mild CTS, patients with severe CTS, and patients with moderate CTS. Analysis of results was done by The Statistical Package for the Social Sciences (SPSS) software (IBM Corp., Armonk, NY). The results showed that out of 300 hypothyroid patients, 20 patients had moderate CTS and 160 patients had mild CTS. These results concluded that a significant percentage of female hypothyroid patients had been diagnosed with CTS syndrome [18].

There are very few studies that clearly identify the nature of the hypothyroidism and CTS association. To evaluate the relationship between hypothyroidism and CTS, Palumbo et al. (2000) conducted a controlled study. They questioned 26 hypothyroid patients about the date of thyroid replacement therapy, the diagnosis of hypothyroidism, and the duration of CTS symptoms. This study also included 24 healthy volunteers as controls. Clinical examination was also performed, which included various tests such as compression test, Phalen's test, Weber 2-point discrimination testing, and electrodiagnostic testing (for diagnosing the compression of the median nerve). The symptoms of CTS were observed in 19 hypothyroid patients. The hypothyroid patients with CTS were also euthyroid [19].

Advanced age, excessive use of the wrists and hands, obesity, thyroid diseases, and pregnancy are some of the risk factors for CTS. Roshanzamir, Mortazavi, and Dabbaghmanesh (2016) conducted a research study to determine the response of hypothyroid patients to surgical decompression of median nerves. This study involved the recruitment of 22 euthyroid and 25 hypothyroid patients. Serum thyroid-stimulating hormone (TSH) levels were used to determine hypothyroidism. The Boston Carpal Tunnel Syndrome Questionnaire (BCTQ) was also provided to the patients. The analyzed results showed a decrease in postoperative BCTQ scores in comparison with preoperative BCTQ. Moreover, patients with euthyroidism showed a decrease in the score of BCTQ as compared to hypothyroid patients. These results concluded that hypothyroidism is associated with postoperative CTS patients as compared to euthyroidism [20].

In Saudi Arabia, there is also a need for such a study that describes the association between CTS and hypothyroidism because the diagnosis is necessary to treat both disorders. Early diagnosis greatly helps in the counseling and surgical treatment of patients for the better management of these disorders.

## Materials and methods

This is a retrospective study, carried out in King Fahad Medical City (KFMC) in Riyadh, Saudi Arabia, in 2020. The study population was recruited from KFMC CTS patients who underwent carpal tunnel release surgery files. Inclusion criteria included any gender, any nationality, and patients who underwent carpal tunnel release surgery during 2015-2020. Patients who have not done carpal tunnel release surgery, and patients who have done carpal tunnel release surgery before 2015 were excluded. The sample size was calculated using the EPI info program (Centers for Disease Control and Prevention in Atlanta, Georgia) based on a 95% confidence interval, 5% margin of error, and the total population of Saudi Arabia. The estimated sample size was 384 and will be adjusted to 422 to compensate for the 10% non-response rate.

Data were collected from the KFMC plastic surgery departments' patients’ files. A convenient non-probability sampling technique was employed to collect the data from the participants. The data were coded, entered, and analyzed using the SPSS version 23. Qualitative data were expressed in the form of numbers and percentages (No. and %). The chi-square (χ2) test will be used to examine qualitative data between two groups. Respective approval of the study was obtained from the Research Ethics Committee at King Fahad Medical City. All participants were volunteers and asked to do their best. All data were kept confidential and used only for research purposes.

## Results

**Figure 1:**
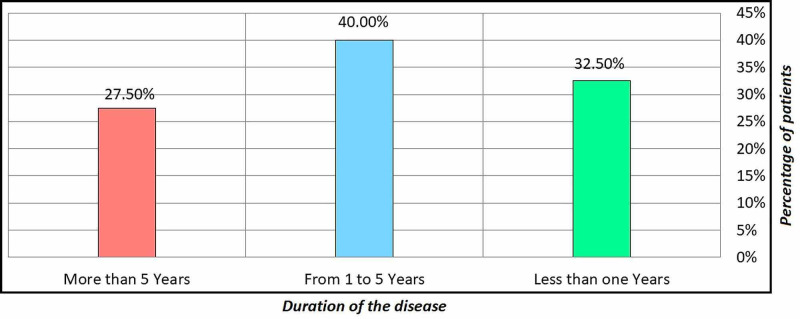
Duration of disease and variable data of the sample

Table 1 shows the frequency distribution of sociodemographic data among CTS release patients. The age of two-thirds (66.3%) of the patients ranged between 46 and 60 years. The predominant gender of our patients (86.3%) was female. In less than half (40.0%) of the patients, their duration of disease ranges between one and five years, and only 27.5% had the disease for more than five years (Figure 1). About one-third (30.0%) of the patient's BMIs were obese class I, and 27.8% were employed. Moreover, the etiology of the patients in the sample were idiopathic.

**Table 1:** Frequency distribution of the sociodemographic data of the sample BMI: body mass index

Variable		Frequency	Percent
Age	From 18 to 30 years	1	1.3%
	From 31 to 45 years	12	15.0%
	From 46 to 60 years	53	66.3%
	More than 61 years	14	17.5%
gender	Male	11	13.8%
	Female	69	86.3%
Duration of disease	Less than one year	26	32.5%
	From 1 to 5 years	32	40.0%
	More than 5 years	22	27.5%
BMI	Normal weight	5	6.3%
	Overweight	23	28.7%
	Obese class I	24	30.0%
	Obese class II	14	17.5%
	Obese class III	8	10.0%
	Obese class IV	4	5.0%
	Obese class V	2	1.5%
Etiology	Idiopathic	80	100%
Total		80	100%

Abnormality and abnormal thyroid among CTS patients

**Figure 2:**
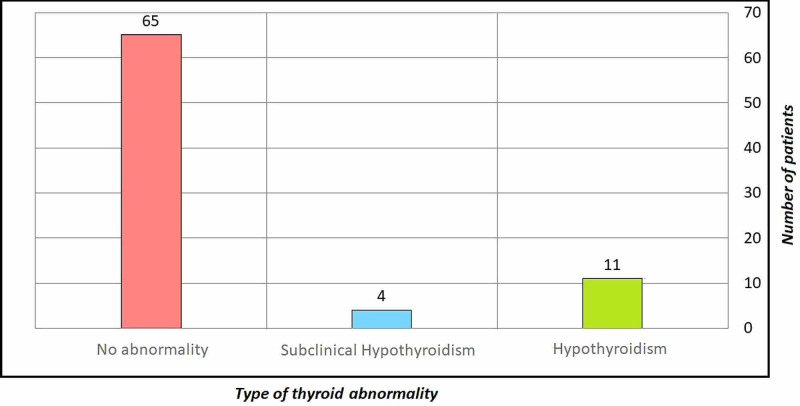
The crossing between the type of abnormality and abnormal thyroid

Table 2 shows the crossing between the type of abnormality and abnormal thyroid among CTS release patients. Data analysis revealed that 15 patients from all patients in the sample suffered from abnormal thyroid. Moreover, 11 of them suffered from hypothyroidism as compared to only four respondents who suffered from the subclinical form of the disease (Figure 2).

**Table 2:** The crossing between the type of abnormality and abnormal thyroid

Abnormal thyroid		yes		no		Total
type of abnormality	Choices	Number	Percent	Number	Percent	
	Hypothyroidism	11	13.8%	0	0.0%	11
	Subclinical	4	5.0%	0	0.0%	4
	No abnormality	0	0.0%	65	81.3%	65
Total		15	18.8%	65	81.3%	80

Prevalence of hypothyroidism among CTS patients at KFMC in Saudi Arabia

**Figure 3:**
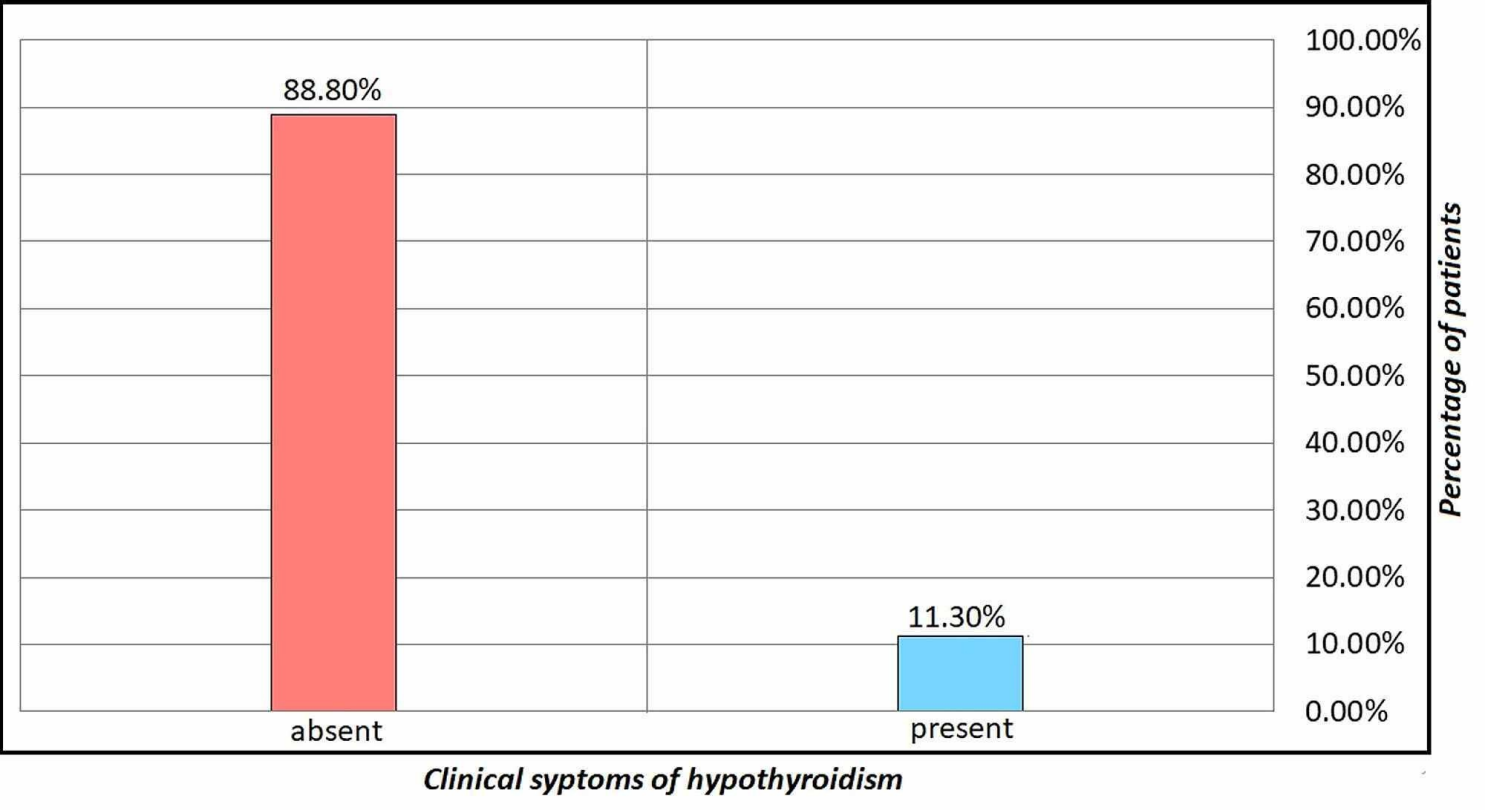
Frequency distribution of clinical symptoms of hypothyroid with CTS CTS: carpal tunnel syndrome

Figure 3 showed the clinical symptoms of hypothyroid with CTS. In most patients (88.8%), clinical symptoms were absent while clinical symptoms were present only in 11.3%.

Frequency distribution of the duration of CTS and nerve condition

Table 3 revealed that the highest frequency of the duration of disease among the patient was from one to five years, with a percentage of 40.0% while the lowest frequency (more than five years) with a percentage of 27.5%. It was observed from the table that the mean was 3.9428 with a standard deviation of 1.05869. The explanation of this finding is that the duration of the disease is from one to five years.

**Table 3:** Frequency distribution of duration of CTS and nerve condition among the study participants CTS: carpal tunnel system

Variable	Choices	Frequency	Percent
Duration of CTS	Less than one year	26	32.5%
	From 1 to 5 years	32	40.0%
	More than 5 years	22	27.5%
Mean ± SD		1.95 ± 0.778	
Nerve condition	Right wrist	23	28.7%
	Left wrist	12	15.0%
	Bilateral	45	56.3%
Mean ± SD		2.275 ± 0.886	

**Figure 4:**
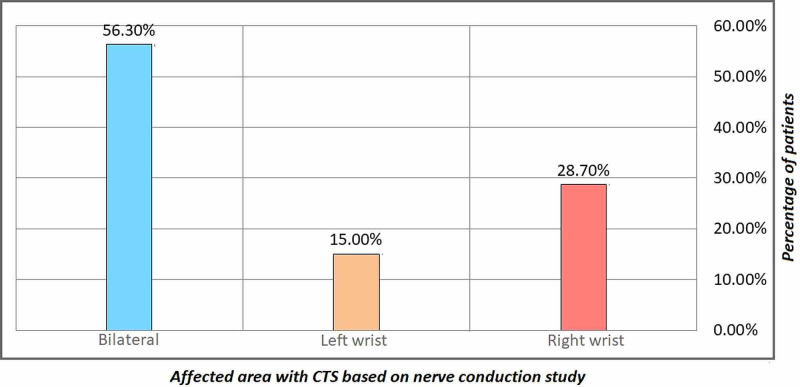
Nerve condition and variable data of the sample

It is clear that the nerve condition among the patient (bilateral) has a percentage of 56.3% while the lowest frequency (left wrist) has a percentage of 15.0%) (Figure 4).

The table also showed a mean of 3.9428 with a standard deviation of 1.05869. The explanation for this finding is that the most observed nerve condition was bilateral.

Relationship between clinical signs and symptoms and nerve conduction

Table 4 showed the relationship between clinical signs and symptoms and nerve conduction. There is no statistically significant relationship between clinical signs and symptoms and nerve conduction in all the patients (p-value = 0.439, which is more than 0.05).

**Table 4:** Relationship between clinical signs and symptoms and nerve conduction

Choices		Clinical signs and symptoms				Test ꭓ²	Sig.
		Absent		Present			
		Number	Percent	Number	Percent		
Nerve conduction	right wrist	22	27.5%	1	1.3%	1.646	0.439
	left wrist	10	12.5%	2	2.5%		
	Bilateral	39	48.8%	6	7.5%		
Total		71	88.8%	9	11.3%		

Relationship between hypothyroidism and duration of CTS

Table 5 shows the relationship between hypothyroidism and the duration of CTS. It is clear there is no relationship between hypothyroidism and the duration of CTS in all the patients because the significant p-value (0.773) is more than 0.05. Four patients had a disease duration of less than one year or one to five years and three patients with a duration of more than five years had hypothyroidism

**Table 5:** Relationship between hypothyroidism and duration of CTS CTS: carpal tunnel syndrome

Choices		Hypothyroid						Test ꭓ²	Sig.
		Yes		No		No abnormality			
		Number	Percent	Number	Percent	Number	Percent		
Duration of the disease	Less than one Years	4	5.0%	2	2.5%	20	25.0%	1.795	0.773
	From 1 to 5 Years	4	5.0%	2	2.5%	26	32.5%		
	More than 5 Years	3	3.8%	0	0.0%	19	23.8%		
Total		11	13.8%	4	5.0%	65	81.3%		

Relationship between the prevalence of Hypothyroidism among CTS patients and other sociodemographic factors

The relationship between hypothyroidism and the sociodemographic factors of the patients is summarized in Table 6. There was no significant association between age and gender in relation to hypothyroidism, as the calculated p-values were recorded as 0.664 and 0.230, respectively. While a significant relationship was observed between BMI and hypothyroidism (p-value = 0.001, which is less than 0.05). Obese patients in class 1 more frequently have hypothyroidism as compared to other BMI index categories.

**Table 6:** Relationship between hypothyroidism and sociodemographic factors

Choices		Hypothyroid						Test ꭓ²	Sig.
		Yes		No		No abnormality			
		N	%	N	%	N	%		
BMI	Normal weight	0	0.0%	1	1.3%	4	5.0%	32.61	0.001
	Overweight	0	0.0%	1	1.3%	22	27.5%		
	Obese class I	5	6.3%	1	1.3%	18	22.5%		
	Obese class II	3	3.8%	0	0.0%	11	13.8%		
	Obese class III	1	1.3%	0	0.0%	7	8.8%		
	Obese class IV	2	2.5%	0	0.0%	2	2.5%		
	Obese class V	0	0.0%	1	1.3%	0	0.0%		
Age	From 18 to 30 years	0	0.0%	0	0.0%	1	1.3%	4.095	0.664
	From 31 to 45 years	1	1.3%	1	1.3%	10	12.5%		
	From 46 to 60 years	6	7.5%	2	2.5%	45	56.3%		
	More than 61 years	4	5.0%	1	1.3%	9	11.3%		
Gender	Male	0	0.0%	0	0.0%	11	13.8%	2.943	0.230
	Female	11	13.8%	4	5.0%	54	67.5%		
Total		11	13.8%	4	5.0%	65	81.3%		

## Discussion

A total of 422 respondents were enrolled in the study; 86.3% were females and the majority were within the age group of 46 - 60 years. Forty percent had CTS for one to five years and 30% were obese class I.

The results of our participants demonstrate that CTS is prevalent in patients with thyroid abnormalities, especially those with hypothyroidism. This is quite similar to a finding in a parallel study conducted in a UK teaching hospital with a sample size of 100. The prevalence of the thyroid problem in their CTS patients was 3% [5], which is almost the exact value that we found (3.5%). Moreover, a meta-analysis study by Shiri (2014) summarized the results of 10 studies; all of them conclude that CTS is strongly related to thyroid abnormality “hypothyroidism” [17].

Our findings were specific, as they expressed the prevalence of thyroid abnormalities in CTS patients among both males and females, as they did in a similar study that specified females as the gender with the more frequent thyroid concerns [18].

CTS could be predicted with many risk factors, such as age, obesity, pregnancy, excessive hand and wrist usage, along with hypothyroidism [20]. In terms of CTS risk factors, our results demonstrate the significant association between BMI and hyperthyroidism (p-value = .001) and explain the relationship of other sociodemographic factors, such as gender, which associate with hypothyroidism, but this relation is not statistically significant. However, gender significantly predicts hypothyroidism as reported by Roshanzamir, Mortazavi, and Dabbaghmanesh (2016) [20].

The results revealed that age distribution and hypothyroidism are associated, with those in the age group of 46 - 60 years more frequently having hypothyroidism symptoms than other younger age groups but this association is not statistically significant (p-value = .664). In the same context, authors of a parallel study in 2016 declare that age significantly predicts hypothyroidism, especially in females over the age of 60 years [7].

Bilateral nerve conduction problems mainly present with overt signs and symptoms. Also, hypothyroidism tends to present in a patient with CTS for one to five years more than those who had the disease for less than one year or more than five years.

## Conclusions

Most patients with thyroid abnormalities present with hypothyroidism and fewer patients with the sub-clinical form. The clinical symptoms of hypothyroidism are mostly absent in patients with CTS. Most patients with CTS have the disease for one to five years and this is not significantly associated with abnormal thyroid. Most patients have bilateral wrist involvement with no apparent symptoms and signs. There is a significant relationship between hypothyroidism and BMI in all the patients (p-value = 0.001).

CTS is painful and can be disabling. Advanced age, excessive use of wrists and hands, obesity, and thyroid diseases are some of the risk factors of CTS. It is important to assess such risk factors to be modified, which, in turn, can prevent the progress of the disease. Clinical symptoms of hypothyroidism are mostly absent in patients with CTS. Therefore, screening for hypothyroidism in patients with CTS can prevent and halt the progress of these disorders, minimize their occurrence, and might be reversible at early stages after appropriate hormone replacement of thyroxine in newly diagnosed patients before considering a surgical approach.
